# On the Veins of Bones

**Published:** 1804-12-01

**Authors:** 


### On the Veins of Bones, by Mr. Dupuytren.

THE veins situated in the interior of the bones and cartilages are but little known, because it is impossible to inject them ; and, in order to find these canals, it is necessary to search for the trunks at their issue from the bones, or to trace them in the substance of the bones. They generally accompany the arteries, which may be rendered visible by injection. In the flat bones they are discovered by elevating the exterior tabula; and in the other bones, by dividing the extremities as well as the middle part of the bones with the anatomical saw in different directions. The action of acids and the combustion facilitate these researches. In dry bones they are seen to arise from the spongy texture with very fine radiculæ, and afterwards to unite under acute angles for forming branches and trunks. These, though contained within the substance of the bone, permit a circulation, which must differ from that which takes place in the soft parts, at least it needs none of the expedients which physiologists appoint for the circulation in general. The veins of bones are hardly visible in infants, but they are very much dilated, and full, in old people. They vary with respect to their number. On the cranium there are generally three or four on each side, which take their direction towards the basis, where they terminate in other trunks, as the exterior veins, in those that accompany the arteriæ meningeæ, and even in the sinus. There are two of them in each vertebra: they open themselves in the sinus of the posterior surface. The veins of the extremities of the long bones and the cartilages pass to the neighbouring veins. Those veins have, under certain circumstances, occasioned mortal hæmorrhagies.

